# Acute and Chronic Effects of Clothianidin, Thiamethoxam and Methomyl on *Chironomus dilutus*

**DOI:** 10.1007/s00128-021-03416-z

**Published:** 2021-12-07

**Authors:** Bryn M. Phillips, Jennifer P. Voorhees, Katie Siegler, Laura McCalla, Peter Meertens, Julie Bower, Ron S. Tjeerdema

**Affiliations:** 1 Marine Pollution Studies Laboratory, Department of Environmental Toxicology, University of California Davis, 34500 Highway One, Monterey, CA 93940 USA; 2 Central Coast Regional Water Quality Control Board, 895 Aerovista Place, Suite 101, San Luis Obispo, CA 93401-7906 USA

**Keywords:** Clothianidin, Thiamethoxam, Methomyl, Neonicotinoid, Carbamate

## Abstract

Organism tolerance thresholds for emerging contaminants are vital to the development of water quality criteria. Acute (96-h) and chronic (10-day) effects thresholds for neonicotinoid pesticides clothianidin and thiamethoxam, and the carbamate pesticide methomyl were developed for the midge *Chironomus dilutus* to support criteria development using the UC Davis Method. Median lethal concentrations (LC50s) were calculated for acute and chronic exposures, and the 25% inhibition concentrations (IC25) were calculated for the chronic exposures based on confirmed chemical concentrations. Clothianidin effect concentrations were 4.89 µg/L, 2.11 µg/L and 1.15 µg/L for 96-h LC50, 10-day LC50 and 10-day IC25, respectively. Similarly, thiamethoxam concentrations were 56.4 µg/L, 32.3 µg/L and 19.6 µg/L, and methomyl concentrations were 244 µg/L, 266 µg/L and 92.1 µg/L. Neonicotinoid effect concentrations compared favorably to previously published 96-h and 14-day LC50 concentrations, and methomyl effect concentrations were within the acute survival range reported for *Chironomus* species and other organisms.

Organism tolerance thresholds for emerging contaminants are vital to the development of water quality criteria. A national water quality criteria methodology was established by the U.S. EPA in 1985 (U.S. EPA 1985), but newer methods have added valuable procedures for developing criteria (TenBrook et al. 2009). More recently, University of California Davis and California’s Central Valley Regional Water Quality Control Board developed a method for the derivation of aquatic life pesticide water quality criteria that utilizes features from previous methodologies to “generate the most flexible and robust criteria” (TenBrook et al. 2010). The development of the University of California Davis Method (UCDM) was the second part of a larger project that also included use of the method to derive criteria for pesticides of concern.

Criteria derivation requires appropriate, high quality ecotoxicological effects data from taxonomically diverse groups of organisms (TenBrook et al. 2010). The UCDM provides a list of data requirements, including physical–chemical data, ecotoxicological data and human health action levels. Data needs in the ecotoxicological category include acute and chronic laboratory exposures with aquatic insects. Acute exposures are defined as lasting up to 96 h with a survival endpoint, whereas chronic exposures include a portion of the organism life cycle and include endpoints such as growth or reproduction.

Three insecticides were chosen based on their levels of concern in California (USA) receiving waters. California Department of Pesticide Regulation currently prioritizes imidacloprid and methomyl for monitoring in waterways receiving agricultural inputs, and recommend clothianidin be included in future monitoring efforts (Main 2019). These insecticides are frequently detected in agricultural watersheds at concentrations exceeding U.S. Environmental Protection Agency (U.S. EPA) aquatic life benchmark values, and are known to cause toxicity to laboratory test organisms (Anderson et al. 2018; Starner and Goh 2012).

The objectives of this study were to not only develop thresholds that could contribute to the development of UCDM criteria, but to provide toxicity threshold values that could be used to interpret ongoing monitoring data. The concentrations of detected insecticides in receiving waters can be compared to LC50 values to determine the contributions of those chemicals to the observed toxicity.

## Methods

All tests were conducted at the University of California, Davis Marine Pollution Studies Laboratory (MPSL). Methods for the acute toxicity test followed U.S. EPA (2002), and methods for the chronic test follow USGS (Ingersoll et al. 2013; Kunz et al. 2017). Rangefinder tests were conducted for each test duration and insecticide. Rangefinder tests consisted of a broad range of concentrations assured to cause mortality on the high end, and were based, in part, on existing published thresholds for *Chironomus* and similar species. The clothianidin and thiamethoxam rangefinder tests were conducted with nominal concentrations ranging between 1 and 200 µg/L. The methomyl rangefinder test was conducted with nominal concentrations ranging up to 5000 µg/L. Rangefinder tests were followed with two definitive tests for each test duration and chemical.

Test solutions were prepared by diluting 10 mg pure standard reference materials (Accustandard, New Haven, CT) in one mL acetone. This solution was added to a one-liter volumetric flask of distilled water, and test concentrations were prepared through further dilution using volumetric flasks and MPSL well water. Toxicity tests consisted of four replicate 300-mL glass beakers for each chemical concentration and four beakers for the negative control consisting of MPSL well water. Approximately 5 mL of clean sand was added to each test container as a substrate for the organisms. Twelve organisms were added to each beaker. Beakers were arranged randomly, and each received 200 mL of test solution. Tests were conducted at 23°C and a photoperiod of 16 h light: 8 h dark.

Egg cases were purchased from Aquatic Bio Systems (Fort Collins, CO), and were ordered to arrive eight to ten days prior to test initiation. Larvae were hatched and acclimated to test temperature and conductivity. Larvae were between second and third instar at test initiation with at least 50% of larvae at third instar, approximately 7 days post hatch. Upon arrival, egg cases were placed in a 250-mL crystalizing dish containing MPSL well water at a temperature that varied by no more than 3°C from transport conditions. At the first sign of hatching, egg cases were carefully transferred to a culture dish prepared with well water and clean sand, and provided aeration. Every 24 h egg cases were transferred into a newly prepared dish until sufficient larvae were acquired, or until the egg cases were spent. Cultures were fed daily and overlying water was renewed every other day. Larval health was monitored daily by observing appearance. If more than 5% of the larvae appear unhealthy during the 48 h prior to the test, the test was rescheduled. Larvae were individually placed into test containers using a clean transfer pipette. Only animals that were healthy and had built tubes were used in the test batch.

Temperature, dissolved oxygen, pH, ammonia, and conductivity were measured at test initiation and termination. Hardness and alkalinity were measured at the beginning of the test. Initial test solutions were sampled at the time of solution preparation. Test solutions were renewed by slowly pouring half of the solution from the container, taking care not to remove or disturb any organisms, and reserving a portion for water quality measurements. Fresh tests solutions were introduced by gently pouring down the side of the beaker to minimize turbulence and stress to the organisms. Water quality was measured on old and new solutions at the time of renewal, and included dissolved oxygen on new samples, and DO, pH, and conductivity on old samples.

Acute test replicates were fed 0.5 mL 4 g/L Tetramin® slurry daily, and chronic tests were fed based on test day (0.5 mL Tetramin® slurry for the first 4 days, 1.0 mL on days 5–7, and 1.5 mL on days 8–10). Food was thoroughly mixed before aliquots were taken, and care was taken to prevent formation of fungal matting on the surface of the sand as that can be a sign of overfeeding.

Tests were terminated by sieving organisms from the sand at the bottom of the exposure chambers. The contents of the beaker were poured through a 500 µm screen, and water was gently sprayed on the sand to expose the surviving larvae in their tubes. Surviving larvae generally had a rich red color and were active, whereas dead larvae had lost their color. In addition to color, larvae that were not moving were assessed under a dissecting microscope to determine mortality. Missing larvae were considered dead. Larvae were counted and discarded for acute tests, but were weighed for chronic tests by thoroughly rinsing and transferring them to a pre-ashed foil packet. Packets were first dried overnight at 60°C, cooled in a desiccator for one hour, and weighed to the nearest 0.0001 g. Packets were then placed into crucibles and ashed in a muffle furnace at 550°C for 2 h, cooled in the desiccator, and reweighed to the nearest 0.0001 g.

All chemical concentrations from the definitive tests were extracted using U.S. EPA method 3510C, and measured using U.S. EPA method 8321. Reporting limits for clothianidin, thiamethoxam and methomyl were 0.4 µg/L, 1.0 µg/L and 0.1 µg/L, respectively. Statistical calculations of rangefinder tests were based on nominal concentrations, and calculations from definitive tests were based on confirmed chemical concentrations using Comprehensive Environmental Toxicology Information System™ (CETIS) software (McKinleyville, CA). Median-lethal concentrations (LC50s) were calculated for 96-h and 10-day survival using trimmed Spearman-Karber analysis, and 25% inhibition concentrations (IC25s) were calculated for 10-day growth using linear interpolation. No observed and lowest observed effect concentrations (NOECs and LOECs) were calculated for all endpoints using analysis of variance (ANOVA) with Dunnett’s tests (α = 0.05). Maximum acceptable toxicant concentration (MATC), a primary component of the UCDM, was calculated from NOEC and LOEC values.

## Results and Discussion

All 96-h and 10-day tests were paired and initiated at the same time. All toxicity tests met test acceptability criteria of > 90% survival in the control with the exception of the first thiamethoxam 96-h test. These results led to conducting a third thiamethoxam definitive test. Most water quality results were within acceptable ranges for the test organism: pH – 6.99–9.58, conductivity – 554–1240 µS/cm, temperature – 22.1–24.0°C, hardness – 128–238 mg/L CaCO_3_ and alkalinity 78–163 mg/L CaCO_3_. Some dissolved oxygen concentrations in the latter portions of the 10-day tests dropped below the recommended 2.5 mg/L threshold (range – 1.04–9.78 mg/L). Low oxygen concentrations occurred in higher pesticide concentrations with zero survival, and were likely the byproduct of surplus food. Reference toxicant tests with potassium chloride were conducted concurrently with definitive tests. Median lethal concentrations from all reference toxicant tests ranged from 3.99 to 6.07 mg/L.

Measured concentrations of clothianidin ranged from 27% to 52% of nominal concentrations. The low recovery of clothianidin in the experimental samples was reflected in the percent recovery of the analytical laboratory control spikes, which ranged from 40.8% to 51%. The UCDM prefers measured concentrations to be within 20% of nominal concentrations. Because analytical laboratory control spike recoveries were less than the recovery limit of 70%, and because measured treatment concentrations of clothianidin were so low, final concentrations were corrected by taking the mean between the measured and nominal concentrations (Raby et al. 2018). Thiamethoxam recoveries were higher and ranged from 62% to 105% of nominal concentrations. Percent recovery of the analytical laboratory control spikes ranged from 72.3% to 95.3% (recovery limit = 70%). Measured concentrations of methomyl ranged from 60% to 76% of nominal concentrations, and were also corrected as described above. Percent recovery of the analytical laboratory control spikes ranged from 67.5% to 77.9% (recovery limit = 23%). Surrogate recoveries of tributylphosphate in the analyses of all three insecticides ranged from 61.3% to 119%, with an acceptable range of 36% to 140%. No insecticides were detected in the method blanks.

Statistical analysis of the clothianidin rangefinder test data produced a 96-h LC50 of 13.9 µg/L, and a 10-day LC50 of 4.45 µg/L (Table 1). Based on these results, definitive tests were conducted with the following concentrations: 1, 2, 5, 10, 25 and 50 µg/L. Based on corrected clothianidin concentrations, two acute tests produced LC50 values of 8.82 and 7.64 µg/L. Corresponding chronic tests produced LC50 values of 3.54 and 3.46 µg/L, and IC25 values of 1.86 and 1.93 µg/L.

**Table 1:** Summary of statistical results for individual clothianidin tests: point estimates and NOEC/LOEC/MATC based on nominal (rangefinder) and corrected (definitive) concentrations

Clothianidin point estimates	96-Hour LC50 (µg/L)	95% LCL	95% UCL	10-Day LC50 (µg/L)	95% LCL	95% UCL	10-Day IC25 (µg/L)	95% LCL	95% UCL
Rangefinder	13.9	11.9	16.3	4.85	4.1	5.72	1.64	NA	1.69
Definitive 1	8.82	7.82	9.94	3.51	3.09	3.98	1.86	1.51	2.03
Definitive 2	7.64	6.64	8.79	3.46	3.06	3.91	1.93	NA	NA

Clothianidin comparison	96-h survival			10-day survival			10-day growth		
	NOEC (µg/L)	LOEC (µg/L)	MATC (µg/L)	NOEC (µg/L)	LOEC (µg/L)	MATC (µg/L)	NOEC (µg/L)	LOEC (µg/L)	MATC (µg/L)
Rangefinder	5	10	7.07	1	5	2.24	1	5	2.24
% Control	100%	68%		100%	64%		99%	4%	
Definitive 1	7.55	17.2	11.4	1.50	3.80	2.39	1.50	3.80	2.39
% Control	65%	4%		100%	49%		112%	5%	
Definitive 2	3.40	6.95	4.86	1.37	3.40	2.16	3.40	> 3.40	NA
% Control	100%	60%		98%	59%		42%	NA	

The UCDM utilizes acute LC50 values for criteria development, but because NOEC/LOEC data are the most widely available for chronic tests, the UCDM utilizes the MATC (TenBrook et al. 2010). The actual no effect concentration would lie between the NOEC and LOEC, therefore the MATC, which is the geometric mean of the NOEC and LOEC, is considered a reasonable estimate. Definitive MATC values were 11.4 and 4.86 µg/L for the 96-h survival endpoint, and 2.39 and 2.16 µg/L for the 10-day survival endpoint. A chronic MATC value (2.39 µg/L) could only be calculated for the 10-day growth endpoint of the first definitive test.

Rangefinder data analyses produced a thiamethoxam 96-h LC50 of 61.2 µg/L, and a 10-day LC50 of 50.9 µg/L (Table 2). Based on these results, definitive tests were conducted with concentration ranges similar to those of the rangefinder: 10, 25, 50, 100 and 200 µg/L. Based on measured thiamethoxam concentrations, two acute tests produced LC50 values of 54.3 and 58.5 µg/L. Three chronic tests produced LC50 values of 34.7, 31.8 and 30.3 µg/L, and IC25 values of 23.8, 17.8 and 17.3 µg/L. MATC values for the three endpoints of the first definitive test were 29.7 µg/L. MATC values for the second definitive test were 50.6 µg/L for the 96-h survival endpoint, and 22.6 µg/L for the 10-day endpoints.

**Table 2:** Summary of statistical results for individual thiamethoxam tests: point estimates and NOEC/LOEC/MATC based on nominal (rangefinder) and measured (definitive) concentrations

Thiamethoxam point estimates	96-Hour LC50 (µg/L)	95% LCL	95% UCL	10-Day LC50 (µg/L)	95% LCL	95% UCL	10-Day IC25 (µg/L)	95% LCL	95% UCL
Rangefinder	61.2	55.9	66.9	50.9	45.4	56.9	28.7	NA	30.7
Definitive 1	54.3	49.1	60.1	31.8	28.2	35.9	17.8	12.2	20.1
Definitive 2	58.5	49.3	69.4	30.3	29.1	31.7	17.3	4.2	29.6

Thiamethoxam comparison	96-h survival			10-day survival			10-day growth		
	NOEC (µg/L)	LOEC (µg/L)	MATC (µg/L)	NOEC (µg/L)	LOEC (µg/L)	MATC (µg/L)	NOEC (µg/L)	LOEC (µg/L)	MATC (µg/L)
Rangefinder	50	100	70.7	25	50	35.4	25	50	35.4
% Control	94%	0%		98%	53%		119%	3%	
Definitive 1	21	42	29.7	21	42	29.7	21	42	29.7
% Control	94%	85%		100%	4%		81%	NA	
Definitive 2	32	80	50.6	16	32	22.6	16	32	22.6
% Control	84%	29%		100%	44%		108%	5%	

Statistical analysis of the methomyl rangefinder test data produced a 96-h LC50 of 242 µg/L, and a 10-day LC50 of 254 µg/L (Table 3). Based on these results, definitive tests were conducted with lower concentration ranges: 50, 100, 250, 500 and 1000 µg/L. Based on corrected methomyl concentrations, two acute tests produced LC50 values of 319 and 280 µg/L. Chronic tests produced LC50 values of 356 and 297 µg/L, and IC25 values of 163 and 58.5 µg/L. The IC25 values are based on replicate data that were quite variable, and in the case of the first definitive test, had an interrupted dose response. MATC values for the survival endpoints of the first definitive test were 309 µg/L (MATC for growth could not be calculated). MATC values all endpoints of the second definitive test were 286 µg/L.

**Table 3:** Summary of statistical results for individual methomyl tests: point estimates and NOEC/LOEC/MATC based on nominal (rangefinder) and corrected (definitive) concentrations

Methomyl point estimates	96-Hour LC50 (µg/L)	95% LCL	95% UCL	10-Day LC50 (µg/L)	95% LCL	95% UCL	10-Day IC25 (µg/L)	95% LCL	95% UCL
Rangefinder	242	213	274	254	221	290	9.07	NA	238
Definitive 1	319	287	355	356	329	386	163	15.1	324
Definitive 2	280	252	312	297	270	327	58.5	32.7	133

Methomyl comparison	96-h survival			10-day survival			10-day growth		
	NOEC (µg/L)	LOEC (µg/L)	MATC (µg/L)	NOEC (µg/L)	LOEC (µg/L)	MATC (µg/L)	NOEC (µg/L)	LOEC (µg/L)	MATC (µg/L)
Rangefinder	100	500	224	100	500	224	100	500	224
% Control	96%	13%		98%	15%		91%	3%	
Definitive 1	220	435	309	220	435	309	435	> 435	NA
% Control	90%	18%		100%	20%		13%	NA	
Definitive 2	205	400	286	205	400	286	205	400	286
% Control	88%	11%		94%	15%		35%	6%	

The neonicotinoid effect concentrations developed in this study compared favorably to previously published 96-h and 14-day LC50 concentrations. Recently developed acute LC50 values for clothianidin and thiamethoxam, calculated from recovery-corrected measured concentrations, ranged from 5.93 to 11.6 µg/L and from 55.3 to 61.9 µg/L, respectively (Maloney et al. 2017; Raby et al. 2018). Although some published methomyl effect concentrations were somewhat dated and mostly based on nominal concentrations, LC50s generated in the current study were within the reported range for some invertebrates, but were lower than those previously reported for *Chironomus*. Acute 96-h LC50s ranged from 47 to 760 µg/L for *Gammarus italicus*, *Daphnia longispina* and *Gammarus pulex* (Aboul-Ela and Khalil 1987; Pantani et al. 1997), and the acute 48-h LC50 for *Chironomus* was reported at 1000 µg/L by Norland et al. (1974).

In addition to supporting the development of water quality criteria, effects concentrations produced in this study will also be used to evaluate monitoring data, particularly in California programs such as the Department of Pesticide Regulation (CDPR) Surface Water Monitoring Program (Main 2019), and the State Water Resources Control Boards Surface Water Ambient Monitoring Program (https://www.waterboards.ca.gov/water_issues/programs/swamp/). Well-developed threshold concentrations can provide insight into the causes of toxicity in laboratory tests, and direct water quality managers in their decision making, particularly in support of Total Maximum Daily Loads (Central Coast Regional Water Quality Control Board 2014), and the implementation of best management practices as outlined in the current central California coastal agricultural order (Central Coast Regional Water Quality Control Board 2021). Approximately half of the clothianidin and thiamethoxam concentrations detected in SWAMP samples between 2018 and 2020 exceeded the 96-h LC50s reported in this study.
